# FASN contributes to ADM resistance of diffuse large B-cell lymphoma by inhibiting ferroptosis via nf-κB/STAT3/GPX4 axis

**DOI:** 10.1080/15384047.2024.2403197

**Published:** 2024-09-30

**Authors:** Xing Zhong, Weiwei Zhang, Weiming Zhang, Nasha Yu, Wuping Li, Xiangxiang Song

**Affiliations:** a Departments of Lymphatic and Hematological Oncology, Jiangxi Cancer Hospital (The Second Affiliated Hospital of Nanchang Medical College), Nanchang, Jiangxi, P. R. China; b JXHC Key Laboratory of Tumor Microenvironment and Immunoregulation (Jiangxi Cancer Hospital), Nanchang, Jiangxi, P. R. China; c Nanchang Medical College, Nanchang, Jiangxi, P. R. China

**Keywords:** FASN, drug resistance, ferroptosis, Nf-κB/STAT3/GPX4 axis, DLBCL

## Abstract

Drug resistance is a critical impediment to efficient therapy of diffuse large B-cell lymphoma (DLBCL) patients. Recent studies have highlighted the association between ferroptosis and drug resistance that has been reported. Fatty acid synthase (FASN) is always related to a poor prognosis. In this study, we investigate the impact of FASN on drug resistance in DLBCL and explore its potential modulation of ferroptosis mechanisms. The clinical correlation of FASN mRNA expression was first analyzed to confirm the role of FASN on drug resistance in DLBCL based on the TCGA database. Next, the impact of FASN on ferroptosis was investigated in vitro and in vivo. Furthermore, a combination of RNA-seq, western blot, luciferase reporter, and ChIP experiments was employed to elucidate the underlying mechanism. The prognosis for patients with DLBCL was worse when FASN was highly expressed, particularly in those undergoing chemotherapy for Adriamycin (ADM). FASN promoted tumor growth and resistance of DLBCL to ADM, both in vitro and in vivo. It is noteworthy that this effect was achieved by inhibiting ferroptosis, since Fer-1 (a ferroptosis inhibitor) treatment significantly recovered the effects of silencing FASN on inhibiting ferroptosis, while Erastin (a ferroptosis inducer) treatment attenuated the impact of overexpressing FASN. Mechanistically, FASN activated NF-κB/STAT3 signaling pathway through phosphorylating the upstream IKKα and IκBα, and the activated STAT3 promoted GPX4 expression by directly binding to GPX4 promoter. FASN inhibits ferroptosis in DLBCL via NF-κB/STAT3/GPX4 signaling pathway, indicating its critical role in mediating ADM resistance of DLBCL.

## 1. Introduction

Diffuse large B-cell lymphoma (DLBCL) is the most commonly diagnosed non-Hodgkin’s lymphoma (NHL) and is known for its aggressive nature, accounting for approximately one-third of all cases.^1^ Immunochemotherapy-based treatment is the predominant treatment modality for DLBCL.^2^ However, it is concerning that following first-line treatment, mainly R-CHOP, 40–50% of DLBCL patients suffer a relapse.^2^ Therefore, it is urgent to explore novel and promising therapeutic targets and treatment strategies to improve the overall survival rate of DLBCL patients.

Since the inception of Warburg’s effect, metabolic rewiring has been widely introduced, revealing the basic dynamics of cancer metabolism.^3^ Except for glucose utilization, tumor cells can also utilize multiple nutrients, including amino acids and fatty acids, to support tumor growth and metastasis. As the sole human lipogenic enzyme that contributes to the de novo synthesis of fatty acids,^4^ fatty acid synthase (FASN) is a key modulator in lipid metabolism and, as such, serves a vital role in the malignant progression of tumors with lipogenic phenotypes.^5^ FASN is a multi-enzyme complex comprising six separate enzymatic grooves, which function together to assemble almost all biological membranes, generate secondary messengers, and produce ATP.^6^ The activity of FASN not only accounts for the cell consumption of NADPH but also for acetyl-CoA,^7^ thereby ensuring the reduced equivalent production, and ultimately, counterbalancing the mitochondrial oxidative stress.^8^ It has been reported that FASN is up-regulated in tumors, including endometrial carcinoma,^9^ lung carcinoma,^10^ and gliomas,^11^ with the exception of lactating breast^12^ and cycling endometrium.^13^ Moreover, the accumulation of FASN always results in drug resistance and poor prognosis,^6,14^ but the underlying mechanism remains poorly understood.

Ferroptosis is an iron-dependent form of necrotic cell death, characterized by unlimited lipid peroxidation and plasma membrane damage.^15^ Triggered by the peroxidation of polyunsaturated fatty acids (PUFA), ferroptosis is mainly determined by the regulation of lipid metabolism. It has been reported that the enrichment of PUFAs is induced by the suppression of FASN expression.^16^ Meanwhile, ferroptosis has been linked to drug resistance in numerous cancers.^17,18^ ADM, a drug used in the first-line chemotherapy regimen for DLBCL, is the main cause of drug resistance in DLBCL patients.^19^ Therefore, we used ADM to examine the impact of FASN-mediated ferroptosis on DLBCL resistance. In this study, we confirmed that the up-regulation of FASN is closely correlated with the drug resistance of DLBCL by inhibiting ferroptosis. Mechanistically, the overexpression of FASN activates the nuclear transcription factor-kappa B (NF-κB)/transcription 3 (STAT3) signaling pathway, which subsequently promotes the expression of glutathione peroxidase 4 (GPX4), and eventually, inhibits ferroptosis.

## 2. Materials and methods

### 2.1. Cell culture

The two DLBCL cell lines (SU-DHL-2 and U2932 cells) obtained from DSMZ (German Collection of Microorganisms and Cell Cultures) were cultured in RPMI-1640 medium (Tecno, Hangzhou, China, L121–500) with 10% FBS (SERANA, Brandenburg, Germany, S-FBS-SA-015) and 1% penicillin–streptomycin (Pen-Strep, Gibco, Semitic, NY, USA 15,140,122) added in a 5% CO_2_ incubator at 37°C.

### 2.2. Transient transfection

The siRNAs (including siR-NC and siR-FASN) used in this study were purchased from Hippo Bio (Huzhou, China) for the construction of FANS-silenced U2932 cells. The sequence of FASN and STAT3 mRNA was synthesized and subcloned into the empty vector pCDH (YouBio, Hunan, China, VT1480) to form the FASN or STAT3 overexpressed plasmid. Once cells reached 70% confluence, siR-NC and siR-FASN were separately transfected into U2932 cells, while pCDH-FASN, pCDH-STAT3, and the empty pCDH plasmid were separately transfected into SU-DHL-2 cells using Lipofectamine 2000 (Invitrogen, Waltham, MA, USA 11,668–019) and Opti-MEN (Gibco, Semitic, NY, USA 31,985–070) according to the manufacturer’s description.

### 2.3. Lentivirus infection

The FASN-overexpressed lentivirus (Lenti-FASN) and the control lentivirus (Lenti-control) were obtained from GeneChem (Shanghai, China). Once reaching 80% confluence, SU-DHL-2 cells were infected with Lenti-FASN or Lenti-control for 24 h. Then, 1 μg/mL puromycin (Solarbio, Beijing, China, P8230) was used to screen FASN stably overexpressed SU-DHL-2 cells.

### 2.4. Xenograft model construction

The 4-week-old mice purchased from Slaccas (Shanghai, China) were subcutaneously injected with FASN-overexpressed or the control SU-DHL-2 cells to establish xenograft mice models. The mice were randomly divided into four groups (*n* = 6): (a) Lenti-control+Control, (b) Lenti-FASN+Control, (c) Lenti-FASN+ADM, and (d) Lenti-FASN+ADM+Erastin. Mice were injected intraperitoneally with 2 mg/kg ADM (MedChemExpress, New Jersey, USA, HY-15142A) twice a week or 20 mg/kg Erastin (MedChemExpress, New Jersey, USA, HY-15763) every two days, as indicated by grouping. Administration continued for 2 weeks. After treatment, mice were euthanized and the xenograft tumor tissues were isolated, and the weight and volume of the tumors were measured and recorded. The experimental processes in this study conform to the requirements of the Guiding Principles for the Breeding and Use of Animals (China).

### 2.5. Western blot (WB)

The total protein of the xenograft tumor tissues, SU-DHL-2 and U2932 cells were extracted utilizing a RIPA lysis buffer (Merck, Darmstadt, Germany, 20–188). Then, the protein concentration was determined by a BCA Protein Assay Kit (Beyotime, Shanghai, China, P0010) and adjusted to a consistent level. Subsequently, protein samples were loaded in SDS-polyacrylamide gel electrophoresis and transferred to the 0.45 µm polyvinylidene fluoride (PVDF) membranes (Millipore, Massachusetts, USA, IPVH00010). To avoid non-specific binding, the membranes were blocked with 5% skim milk prepared with TBST buffer solution. Then, the membranes were incubated with the primary antibodies at 4°C and the corresponding secondary antibodies at room temperature in succession. After three washes with TBST, the protein bands were visualized utilizing an enhanced chemiluminescence (ECL) kit (Biosharp, Hefei, China, BL520B). The antibodies used for WB are shown in Table 1.

**Table 1.** Information for antibodies.

Name	Brand	Number	Dilution rate	Applications
β-actin	CST	4970T	1:5000	WB
FASN	Proteintech	10624-2-AP	1:5000	WB, IHC
GPX4	Abclonal	A11243	1:2000	WB, IHC
4-HNE	Abcam	ab46545	1:2000	WB, IHC
IKKα	CST	11930T	1:1000	WB
p-IKKα	CST	2697T	1:2000	WB
IκBα	CST	4814T	1:1000	WB
p-IκBα	CST	2859T	1:2000	WB
NFκB	CST	8242T	1:1000	WB
p-NFκB	CST	3033T	1:2000	WB
STAT3	CST	4904T	1:1000	WB
p-STAT3	CST	9145T	1:1000	WB

### 2.6. MTT assay

The cell proliferation of SU-DHL-2 and U2932 cells was detected by the MTT assay. Cells were first seeded into the 96-well plates at a density of 4 × 10^3^ cells/well and cultured in an incubator for 0, 24, 48, and 72 h. Then, 1 mg/mL MTT was added to the plate (50 μL per well) and cells were incubated for 3 h at 37°C. Following the addition of DMSO into the plate (150 μL per well), the relative cell proliferation was determined using a spectrophotometer (Molecular Device, Silicon Valley, CA, USA) at an absorbance of 570 nm.

### 2.7. Flow cytometry

The flow cytometry was performed to examine the total ROS and lipid ROS of SU-DHL-2 and U2932 cells. Firstly, DCFH-DA (Beyotime, Shanghai, China, S0033S, for total ROS) and BODIPY 581/591 C11 (Abclonal, Wuhan, China, RM02821, for lipid ROS) were diluted using the serum-free culture medium (both diluted to 2 µmol/L). Cells were cultured until they reached 80% confluence, collected and then separately co-incubated with diluted DCFH-DA and BODIPY 581/591 C11 for 30 min at 37°C. A flow cytometer (Invitrogen, Waltham, MA, USA, Attune NxT) was utilized to detect the level of ROS.

### 2.8. Immunohistochemistry (IHC)

The IHC assay was carried out to detect the expression of FASN, GPX4, and 4-HNE in xenograft mice models. The tumor tissues were first paraffin-embedded and sliced into sections approximately 5 μm thick. Then sections were successively soaked in xylene for deparaffinization and in different gradients of ethanol for rehydration. Shortly, slices were placed in citrate buffer (Servicebio, Wuhan, China, G1202) and 3% H_2_O_2_ in succession for antigen retrieval and endogenous peroxidase removal. Subsequently, the sections were incubated in primary antibodies specific for FASN, GPX4, and 4-HNE for all night, and in corresponding secondary antibodies for 1 h. After three times washes, DAB solution (Servicebio, Wuhan, China, G1212) and hematoxylin (Servicebio, Wuhan, China, G1004) were used for staining and counterstaining, respectively. The results were observed under a microscope.

### 2.9. Terminal deoxynucleotidyl transferase-mediated dUTP-biotin nick end labeling (TUNEL) assay

The TUNEL assay was performed to examine the apoptosis of xenograft tumor tissues. The tumor tissues were paraffin-embedded and sliced into sections approximately 5 μm thick. Then the slices were stained using a TUNEL apoptosis assay kit (Abcam, Cambridge, UK, ab66110) in accordance with the producer’s direction. The results were observed and photographed under a microscope.

### 2.10. Quantitative real-time PCR (qRT-pcr)

The total RNA was extracted from FASN-overexpressed and control SU-DHL-2 cells using a TRIzol Reagent (Thermo Fisher, Waltham, MA, USA 15,596,026). Then, the purity and concentration of the RNA were examined by a spectrophotometer (Molecular Device, Silicon Valley, CA, USA). RNA was transformed into cDNA through reverse transcription using a cDNA synthesis kit (Takara, Kyoto, Japan, 6215B). Shortly, the PCR reaction mixture (total 10 μL) was prepared with SYBR, cDNA template, and the primers. Then, qRT-PCR was performed with the ABI 7500 Real-Time PCR System (Thermo Fisher, Waltham, MA, USA). The mRNA expression of GPX4 was quantified utilizing the 2^−ΔΔCt^ method. β-actin as an internal control. The primers of GPX4 and β-actin are shown in Table 2.

**Table 2.** Sequences of primers.

Name	F (5’-3’)	R (5’-3’)
β-actin	AGCGGGAAATCGTGCGTG	CAGGGTACATGGTGGTGCC
GPX4	GGAAGCAGGAGCCAGGGAGT	AGCCGTTCTTGTCGATGAGGA

### 2.11. Bioinformatics

Based on the TCGA database, we generated the survival curves using GraphPad Prism 6.0 and analyzed the relevance between FASN mRNA level and the multiple clinical parameters of DLBCL. Besides, we extracted the total RNA of FASN overexpressed SU-DHL-2 cells. Shortly, the RNA-seq was performed by LC-BIO (Hangzhou, China) for the differentially expressed genes (DEGs) detection, then the heat map and the volcano plot were generated utilizing the R version 3.6.3. On the basis of the sequencing results, Gene Ontology (GO) and Kyoto Encyclopedia of Genes and Genomes (KEGG) analysis were implemented to determine the biological processes and pathways in which the DEGs were enriched.

### 2.12. Dual-luciferase reporter assay

The Dual-luciferase reporter assay was implemented to examine the binding relationship between STAT3 and the GPX4 promoter. The fragments of the 3’UTR region of GPX4 containing the potential-binding site of STAT3, as well as their mutant sequences, were amplified and cloned into the empty PGL3 vector (Promega, Madison, WI, USA) to form the PGL3-GPX4 wild-type (WT) and PGL3-GPX4 mutant (MUT) reporter plasmid. These plasmids and the blank PGL3 vector were separately transfected into SU-DHL-2 cells. Then, the luciferase activity was determined with the Dual-Luciferase® Reporter Assay System (Promega, Madison, WI, USA, E1910) in accordance with the manufacturer’s direction.

### 2.13. Chromatin immunoprecipitation (ChIP)

The CHIP experiment was carried out utilizing the ChIP-Seq High Sensitivity Kit (Abcam, Cambridge, UK, ab185908) according to the manufacturer’s instructions. After fixation with 1% formaldehyde, FASN overexpressed SU-DHL-2 and the control cells were lysed and ultrasonicated on ice. Then, the suspension was centrifuged for 10 min by 14,000 rpm at 4°C, and the supernatant was transferred to a new centrifugal tube. Shortly, the anti-STAT3 primary antibody and protein A/G MagPoly Beads were successively added to the supernatant for immunoprecipitation. The precipitate was decrosslinked to obtain the DNA fragment. Then, DNA was purified for the subsequent qRT-PCR experiment.

### 2.14. Statistical analysis

The experiments in this study were repeated at least three times. Representative data were analyzed by GraphPad Prism 6.0. Statistical analysis for comparison between the two groups was *t*-test and that for comparison among multiple groups was one-way ANOVA. *p* < .05 was considered statistically significant.

## 3. Results

### 3.1. FASN level was closely associated with survival rate and chemoresistance in DLBCL

**Figure 1.**
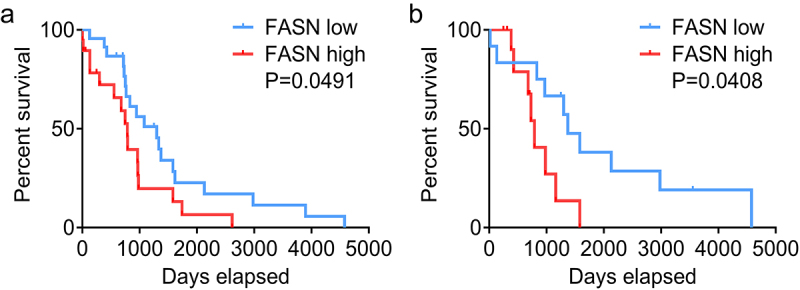
The FASN level was related to the survival and chemoresistance of DLBCL patients. (a) The relevance between FASN mRNA level and the overall survival of DLBCL patients. The threshold for categorizing the cases as high or low expression was set at FASN>10, resulting in a division of 24 cases with high expression and 20 cases with low expression. (b) The correlation between FASN mRNA level and the survival of DLBCL patients undergoing chemotherapy. The threshold for categorizing the cases as high or low expression was set at FASN>10, resulting in a division of 12 cases with high expression and 12 cases with low expression.

Given that the overexpressed FASN served as the molecular marker of unfavorable prognosis and malignant progression in multiple cancers,^6^ we sought to investigate its potential impact on the development and prognosis of diffuse large B-cell lymphoma (DLBCL). To explore the role of FASN in DLBCL, we first analyzed the clinical relevance of the FASN mRNA level in DLBCL based on the TCGA database. As shown in Table 3, there is a statistically significant correlation between FASN expression and the clinical staging of DLBCL, with elevated levels predominantly observed in advanced disease stages. Furthermore, we also found that patients with higher FASN expression exhibited a poorer prognosis (Figure 1a). More importantly, this gap was more obvious in patients undergoing chemotherapy (Figure 1b), suggesting a potential role for FASN in mediating resistance to chemotherapeutic agents in DLBCL.

**Table 3.** Clinical relevance of FASN mRNA expression in DLBCL from TCGA database.

Character	Level	Low expression of FASN	High expression of FASN	P
Age	<60	29.4%	23.5%	.228
	≥60	14.7%	32.4%	
Gender	Male	17.6%	29.4%	.537
	Female	26.5%	26.5%	
B symptoms	NO	41.2%	41.2%	.336
	YES	2.9%	14.7%	
Ann Arbor Stage	I	17.6%	5.9%	.019
	II	20.6%	23.5%	
	III	2.9%	8.8%	
	Ⅳ	2.9%	17.6%	
Extranodal involvement	NO	26.9%	29.4%	.732
	YES	17.6%	26.5%	

### 3.2. FASN mediated the drug resistance of DLBCL to ADM by inhibiting ferroptosis

**Figure 2.**
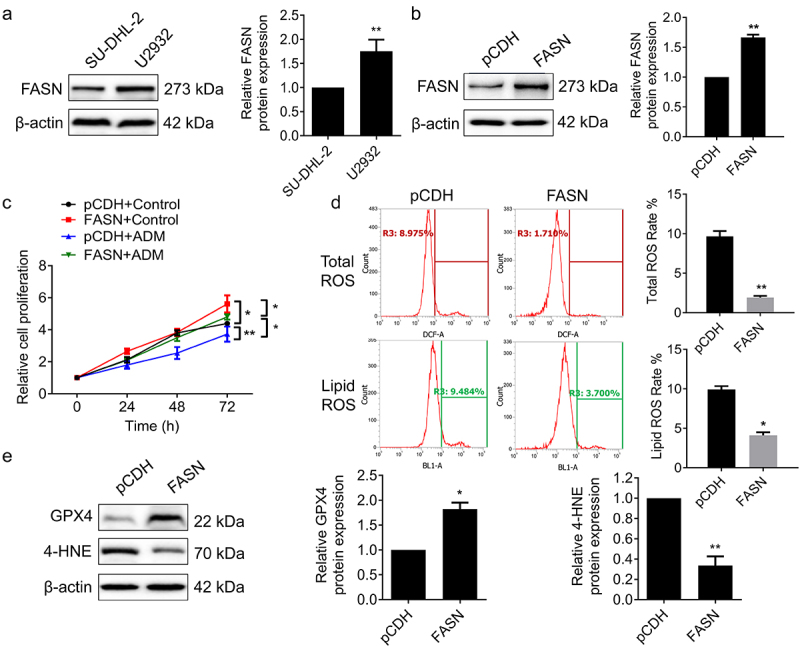
FASN inhibited the anti-tumor effect of ADM and suppressed ferroptosis. (a) The expression of FASN in SU-DHL-2 and U2932 cells. (b) SU-DHL-2 cells were transfected with pCDH-FASN or the empty pCDH vector, the overexpressing efficiency of SU-DHL-2 cells was verified by WB. (c) The effect of overexpression of FASN and ADM treatment (100 nM, 24 h) on the proliferation in SU-DHL-2 cells was determined by the MTT assay. (d) The total ROS and lipid ROS levels of FASN overexpressed and the control SU-DHL-2 cells were examined by flow cytometry. (e) The expression of GPX4 and 4-HNE in SU-DHL-2 cells was detected by WB. β-actin as the endogenous control. Data from at least three repeated experiments. *p <.05, **p <.01.

The expression level of FASN was initially examined in both SU-DHL-2 and U2932 cells. The results demonstrated that FASN was highly expressed in U2932 cells and lowly expressed in SU-DHL-2 cells (Figure 2a). Therefore, to investigate the role of FASN and facilitate loss- and gain-function experiments, we elected to overexpress FASN in SU-DHL-2 cells and knockdown FASN in U2932 cells for subsequent experiments. Subsequently, we constructed FASN-overexpressed SU-DHL-2 cell lines (Figure 2b). ADM (Doxorubicin), a chemotherapeutic agent commonly used in the treatment of lymphoma, was selected to validate the effect of FASN on survival and chemoresistance as previously described. The results demonstrated that the overexpression of FASN facilitated cell proliferation and inhibited the anti-tumor effect of ADM in SU-DHL-2 cells (Figure 2c). According to reports, tumorigenesis is frequently accompanied by abnormalities in lipid metabolism, and rapid cancer progression makes it necessary for lipid metabolism to proceed in the trend of synthetic.^20^ As the key enzyme of fatty acid de novo synthesis, FASN has been more and more studied in the resistance of diverse chemotherapeutic drugs.^21^ Given the pivotal role of ferroptosis in the lipid metabolism pathway and the efficacy of targeting ferroptosis or ferroptosis-related pathways in combating tumor drug resistance,^22^ we speculated that the impact of FASN on drug resistance in DLBCL might involve the regulation of ferroptosis. Ferroptosis is a non-apoptotic form of cell death closely associated with intracellular accumulation of free iron and increased levels of reactive oxygen species (ROS).^23^ During ferroptosis, iron overload within cells leads to lipid peroxidation and membrane damage, ultimately resulting in cell death.^23^ Moreover, elevated ROS levels can promote ferroptosis through multiple pathways.^23,24^ Therefore, we investigated the impact of FASN on intracellular ROS levels and markers associated with ferroptosis. The results demonstrated that overexpression of FASN decreased total ROS and lipid ROS levels (Figure 2d), upregulated the expression of GPX4, and downregulated the expression of 4-HNE (Figure 2e). These findings indicated that overexpression of FASN could inhibit ferroptosis.

**Figure 3.**
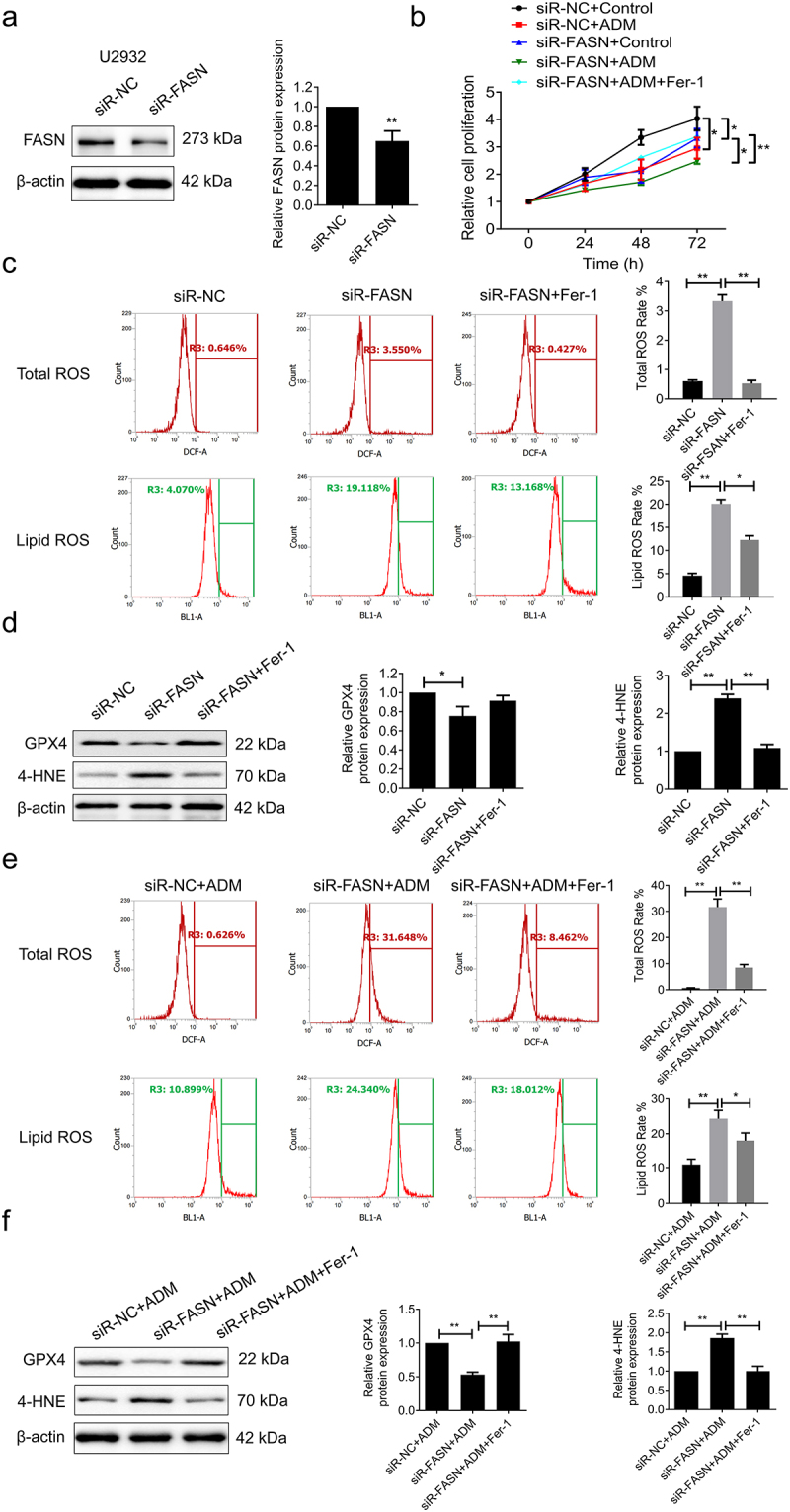
Knockdown of FASN enhanced the anti-tumor effect of ADM by promoting ferroptosis. (a) The silencing efficiency of FASN in U2932 cells was verified by WB. (b) U2932 cells were transfected with siR-FASN or siR-nc after ADM treatment, combined with ferrostatin-1 (fer-1, the ferroptosis inhibitor) treated or not. ADM: 100 nM, 24 h; fer-1: 20 μM 24 h. The cell proliferation in U2932 cells was determined by the MTT assay. (c) The total ROS and lipid ROS levels of the corresponding control and silenced FASN with fer-1 treated or not were examined by flow cytometry. (d) The expression of GPX4 and 4-HNE was detected by WB. (e) The levels of total ROS and lipid ROS in FASN silenced and the control U2932 cells treated with or without ADM were detected by the flow cytometry. (f) The GPX4 and 4-HNE expression was determined by WB. β-actin as the endogenous control. Data from at least three repeated experiments. *p <.05, **p <.01.

To further validate the role of FASN in regulating ferroptosis and drug resistance, FASN was knocked down in U2932 cells for subsequent experiments (Figure 3a). The results of the cell proliferation assays demonstrated that knockdown of FASN inhibited tumor cell proliferation and enhanced the anti-tumor effect of ADM. However, this inhibition of proliferation was significantly attenuated after treatment with Ferrostatin-1 (Fer-1), a ferroptosis inhibitor (Figure 3b). Corresponding to Figure 2d-e, knockdown of FASN increased intracellular total ROS and lipid ROS levels, downregulated the expression of GPX4, and upregulated the expression of 4-HNE, suggesting that knockdown of FASN promotes ferroptosis (Figure 3c-d). Conversely, Fer-1 treatment reversed these effects (Figure 3c-d). Furthermore, knockdown of FASN enhanced the ferroptosis-promoting effect of ADM, which was reversed by Fer-1 treatment (Figure 3e-f). These findings suggested that knockdown of FASN strengthened the anti-tumor efficacy of ADM by facilitating ferroptosis.

### 3.3. FASN promoted tumor growth and resistance of DLBCL to ADM by inhibiting ferroptosis in vivo

**Figure 4.**
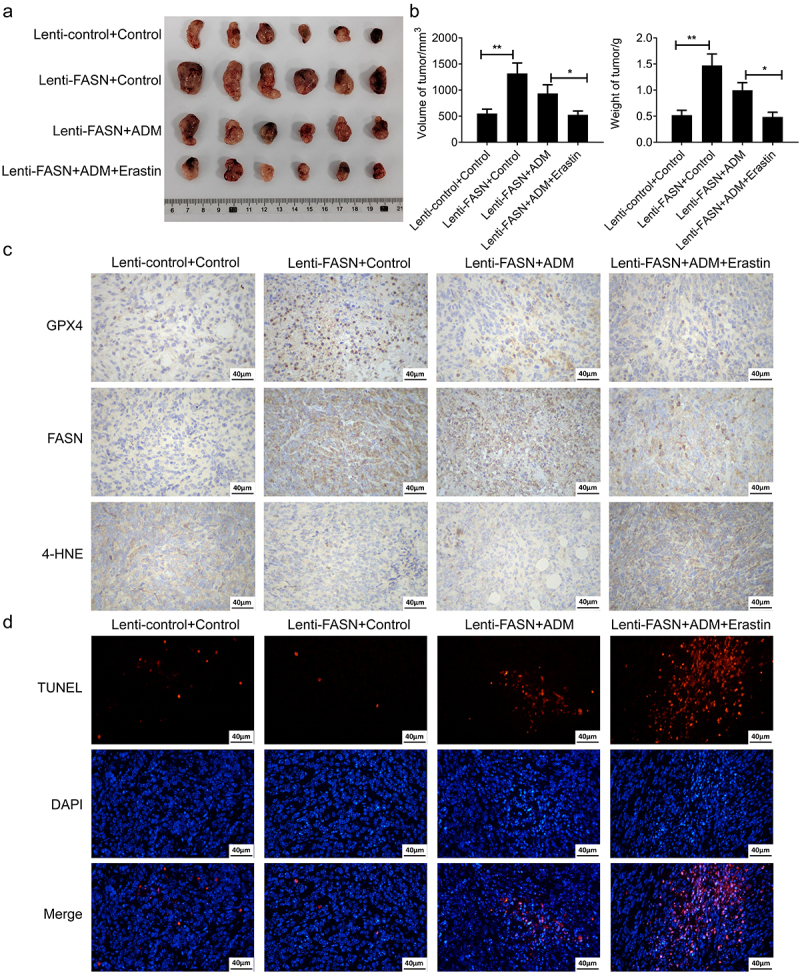
FASN promoted tumor growth and resistance of DLBCL to ADM by inhibiting ferroptosis *in vivo*. The mice (*n* = 6) were injected with FASN overexpressed or the control SU-DHL-2 cells subcutaneously to establish xenograft mice models. The mice were randomly divided into 4 groups, and were injected intraperitoneally with 2 mg/kg ADM twice a week or 20 mg/kg erastin every two days, as indicated by grouping. (a) The xenograft tumor tissues were isolated from the mice. (b) The weight and volume of the tumors were recorded and quantified. (c) The expression of GPX4, FASN, and 4-HNE in tumor tissues was validated by the IHC experiment. (d) The cell apoptosis of tumor tissues was detected by the TUNEL assay. Data from at least three repeated experiments. *p <.05, **p <.01.

To further validate the effect of FASN on tumor growth promotion and resistance to ADM via ferroptosis *in vivo*, the xenograft tumor models were constructed using SU-DHL-2 cells overexpressing FASN. The results demonstrated that overexpression of FASN promoted tumor growth (Figure 4a-b). Furthermore, the overexpression of FASN resulted in the upregulation of GPX4 and the downregulation of 4-HNE in tumor tissues, indicating an inhibition of ferroptosis (Figure 4c). The TUNEL assay also corroborated that the overexpression of FASN inhibited apoptosis in tumor tissues (Figure 4d). Importantly, Erastin (a ferroptosis inducer) reversed the aforementioned effects of FASN and enhanced the anti-tumor efficacy of ADM (Figure 4a-d). To sum up, the experiments *in vitro and vivo* suggested that FASN facilitated the drug resistance of tumor cells to ADM in DLBCL via inhibiting ferroptosis, at least partially.

### 3.4. FASN inhibited ferroptosis by upregulating the GPX4 expression

**Figure 5.**
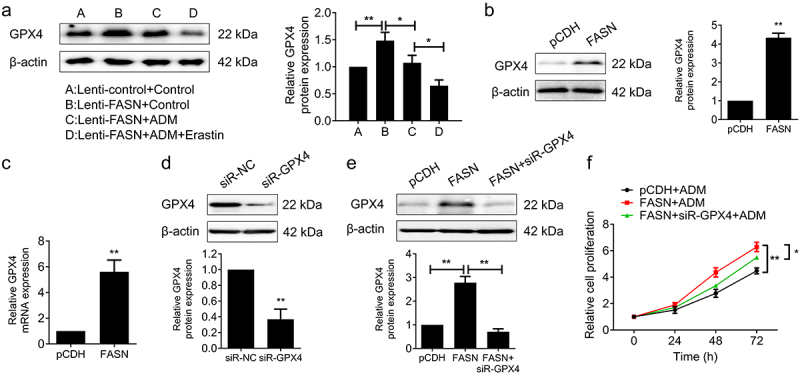
FASN inhibited ferroptosis by upregulating the GPX4 expression. (a) The WB experiment was applied to detect the GPX4 expression of subcutaneous xenograft tumors, and the quantization results are presented as a bar chart on the right. (b&c) the GPX4 expression levels of FASN overexpressed and the control SU-DHL-2 cells were detected respectively by (b) WB and (c) qRT-pcr. (d) SU-DHL-2 cells were transfected with siR-GPX4 or siR-nc, and the silencing efficiency was verified by WB. (e) FASN overexpressed SU-DHL-2 cells were transfected with siR-GPX4 or not, then the GPX expression was assessed by WB. (f) The cell proliferation of SU-DHL-2 cells with ADM treatment (100 nM, 24 h) was detected by the MTT assay. β-actin as the endogenous control. Data from at least three repeated experiments. *p <.05, **p <.01.

The above work verified that FASN promoted the drug resistance to ADM in DLBCL through ferroptosis, but the underlying mechanism needs further exploration. GPX4 is a pivotal regulatory factor in ferroptosis, the inhibition of its functions contributes to lipid peroxidation and the formation of lipid ROS, which results in the occurrence of ferroptosis.^25^ In other words, the upregulation of the GPX4 expression conduces to inhibiting ferroptosis. Thus, we wondered whether FASN could inhibit ferroptosis by regulating the GPX4 expression. The results revealed that the overexpression of FASN resulted in the upregulation of GPX4 expression in Figure 4 tumor tissues (Figure 5a). Consistent results were observed in WB assays conducted on SU-DHL-2 cells compared to experiments *in vivo* (Figure 5b). In addition, the overexpression of FASN also increased GPX4 mRNA levels (Figure 5c). To further confirm that FASN acts through regulating GPX4, we verified the knockdown efficiency of GPX4 and constructed SU-DHL-2 cell lines overexpressing FASN with knockdown of GPX4 (Figure 5 d-e). The results demonstrated FASN-promoted cell proliferation under ADM treatment could be reverted by the silenced GPX4 (Figure 5f). It could be concluded from the results that FASN inhibited ferroptosis by upregulating the GPX4 expression, which in turn promoted cell proliferation and enhanced the resistance to ADM.

### 3.5. FASN inhibited ferroptosis via nf-κB/STAT3/GPX4 axis

**Figure 6.**
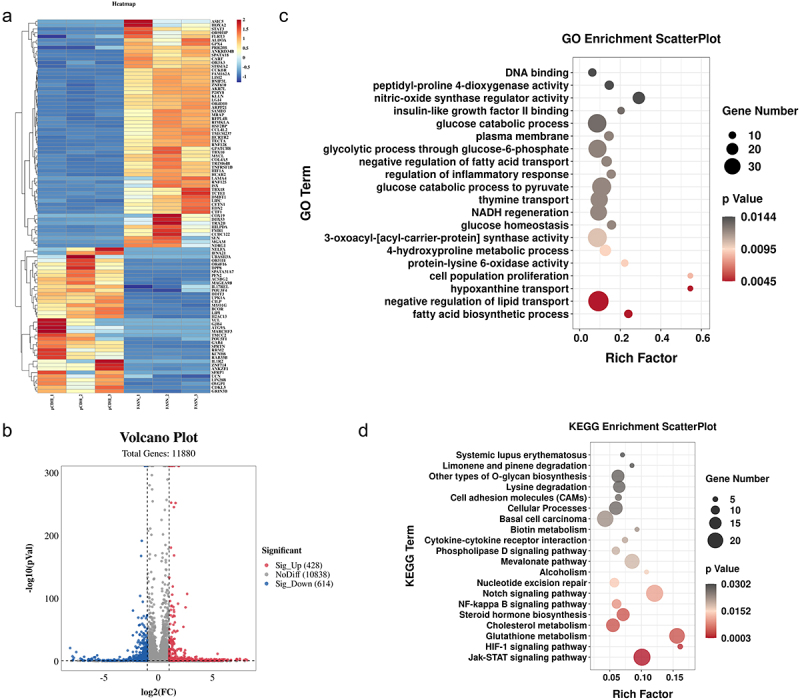
DEGs in FASN overexpressed SU-DHL-2 cells were enriched in nf-κB and Jak-stat signaling pathways. (a&b) the total RNA of FASN overexpressed SU-DHL-2 cells was extracted for transcriptomic sequencing, and the volcano plot presented the differentially expressed genes (DEGs). (c&d) GO and KEGG enrichment analysis was carried out to determine the biological processes and signaling pathways in which DEGs were enriched.

**Figure 7.**
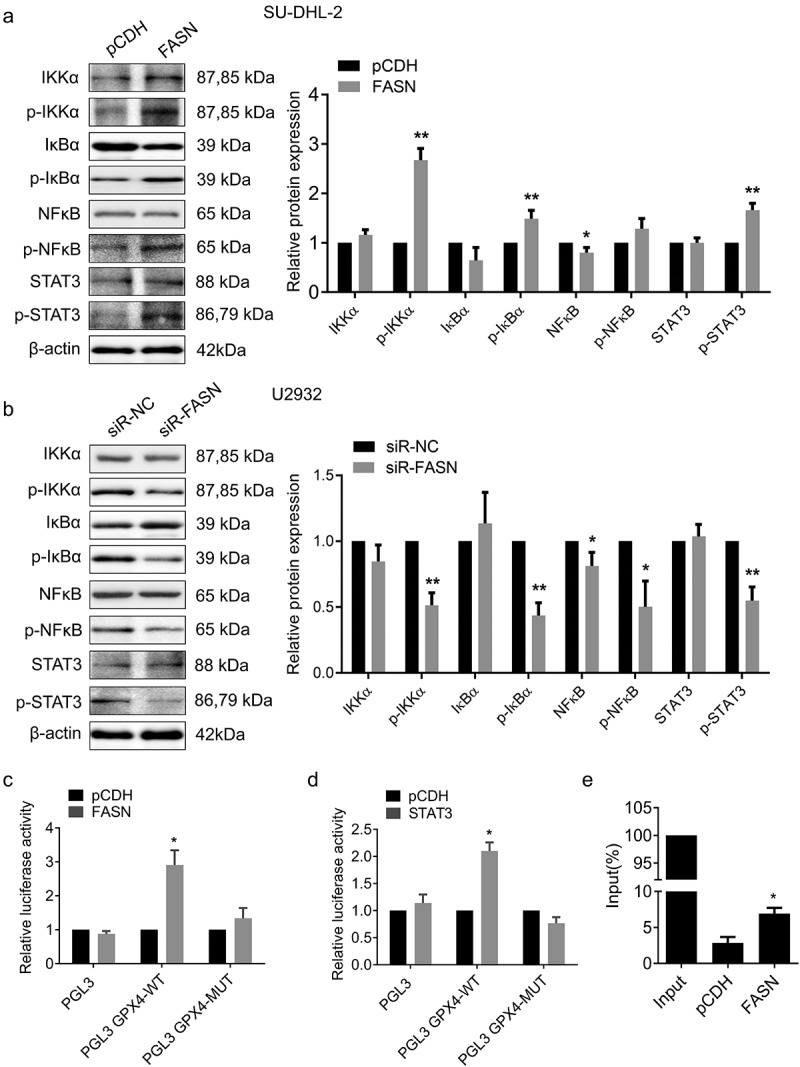
FASN facilitated the binding of STAT3 to the GPX4 promoter. (a&b) the expression of nf-κB and STAT3 signaling pathway-related proteins in (a) FASN overexpressed and the control SU-DHL-2 cells, and that in (b) FASN silenced and the control U2932 cells were determined by WB. β-actin as the endogenous control. (c&d) the potential binding site of STAT3 to the GPX4 promoter was mutated for dual-luciferase reporter assay to determine the influence of (c) FASN or (d) STAT3 on luciferase activity. (e) The regulation of the binding relationship between STAT3 and the GPX4 promoter by FASN was verified using CHIP experiments. Data from at least three repeated experiments. *p <.05, **p <.01.

To further explore the underlying mechanism, RNA-seq was utilized to identify the differentially expressed genes (DEGs) in FASN overexpressed SU-DHL-2 cells (Figure 6a). The results presented 428 up-regulated genes and 614 down-regulated genes (Figure 6b). Subsequent Gene Ontology (GO) enrichment analysis indicated that these DEGs were enriched in fatty acid synthesis metabolism and glutathione metabolism (Figure 6c). Kyoto Encyclopedia of Genes and Genomes (KEGG) analysis pointed out that DEGs were mainly involved in the NF-κB and Jak-STAT signaling pathways (Figure 6d). NF-κB and STAT3 are recognized as pivotal regulatory factors in tumorigenesis, orchestrating processes such as proliferation, survival, and invasion of tumor cells.^26^ The NF-κB and STAT3 signaling pathways are frequently cross-influenced, forming a complex regulatory network.^26,27^ This interaction enhances their roles in cancer and leads to the development of complex oncogenic behaviors such as therapy resistance and increased metastatic capacity.^26,28,29^ Moreover, it has been demonstrated that NF-κB and STAT3 signaling pathways are associated with ferroptosis.^30^ Thus, we wondered whether FASN could facilitate the GPX4 expression through NF-κB and STAT3 signaling transduction. Indeed, FASN facilitated the activation of NF-κB and STAT3 signaling pathways (Figure 7a-b). Subsequently, potential-binding sites between STAT3 and the GPX4 promoter were predicted using the database and subjected to mutation. The results demonstrated that the overexpression of FASN or STAT3 significantly increased luciferase activity; however, no notable changes were observed in cells with mutated GPX4 promoter (Figure 7c-d). Furthermore, the overexpression of FASN facilitated the binding of STAT3 to the GPX4 promoter (Figure 7e). Taken together, we could conclude that the inhibitory impact of FASN on ferroptosis was achieved via NF-κB/STAT3/GPX4 axis, thereby facilitating tumor growth and resistance to ADM in DLBCL.

## 4. Discussion

Refractory DLBCL induced by drug resistance is a significant challenge in improving the prognosis of DLBCL patients. Given the close correlation between metabolism, including lipid metabolism of tumor cells, and drug resistance,^31^ novel anti-tumor targets related to metabolism are needed to overcome the therapeutic challenges of DLBCL. FASN serves as a critical rate-limiting enzyme in the synthesis of fatty acids and is involved in the therapeutic resistance and the progression of tumors, thereby emerging as a promising target in DLBCL.^6^ In our study, based on the clinical data from the TCGA database, we confirmed that FASN was highly expressed in advanced tumors, and it was negatively correlated with the survival rate of DLBCL patients, including those undergoing chemotherapy. These demonstrated that the chemotherapeutic resistance of DLBCL patients was associated with FASN, at least partially. Indeed, this study proved that FASN strengthens chemotherapeutic resistance mainly through ferroptosis in DLBCL. Overexpression of FASN significantly decreased the total and lipid ROS levels, down-regulated 4-HNE expression, yet up-regulated the GPX4 expression in ADM-treated DLBCL cells, SU-DHL-2. Conversely, knocking FASN down exhibited opposite effects on those ferroptosis-related biomarkers, which could be reversed by Fer-1, the ferroptosis inhibitor. These findings confirmed the indispensable role of FASN in regulating ferroptosis to induce drug resistance in DLBCL.

Distinct from autophagy, necrosis, and apoptosis, ferroptosis is a highly iron-dependent form of cell death and is typically modulated by GPX4.^32^ GPX4, the key mediator of ferroptosis, is deeply involved in the reduction of phospholipid hydroperoxides, the accumulation of which leads to cell membrane damage and eventual ferroptosis.^33^ Importantly, it has been widely reported that the regulation of ferroptosis and ferroptosis-related signaling pathways contribute to enhancing the efficacy of anti-tumor therapy, and even overcoming the drug resistance.^34–36^ For example, curcumin analogs have been proven to reverse temozolomide resistance in glioblastoma, based on the fact that their induced ubiquitination of the androgen receptor suppresses GPX4.^37^ Similarly, the oxaliplatin resistance of colorectal cancer can be interrupted by inactivating the KIF20A/NUAK1/PP1β/GPX4 pathway.^38^ In the present study, we also found that FASN-mediated ADM resistance was addressed through ferroptosis via canonical GPX4-regulated pathway, as evidenced by GPX4 knockdown significantly reversed FASN-promoted cell proliferation. Therefore, we tried to elucidate the underlying mechanism by which FASN regulates the expression of GPX4.

Based on the bioinformatics analysis, we found that processes related to ferroptosis, including lipid biosynthesis and transport and plasma membrane integrity, were associated with FASN. Moreover, the overexpression of FASN induced the enrichment of DEGs in the NF-κB and Jak-STAT signaling pathways. Indeed, our subsequent WB experiments proved that the overexpression of FASN resulted in the activation of NF-κB and STAT3 signaling pathways, which means that FASN-suppressed ferroptosis was closely related to the activation of the two pathways above. Furthermore, we identified the binding site of STAT3 on the GPX4 promoter, indicating the mechanism underlying by which FASN negatively regulated ferroptosis in DLBCL. Specifically, the elevated FASN activated NF-κB/STAT3 signaling, which subsequently targeted GPX4 to inhibit ferroptosis.

Both NF-κB and STAT3 are transcription factors associated with the regulation of inflammatory responses.^39,40^ Although literature supported their involvement in ferroptosis,^41,42^ reports on the two transcription factors-regulated ferroptosis engaging in the progression of DLBCL are scarce. In addition, the mechanisms by which NF-κB and STAT3 regulate ferroptosis are controversial. Li *et al*. have proved that the phosphorylated NF-κB up-regulates the expression of GPX4 to inhibit ferroptosis in polycystic ovary syndrome,^43^ while another study revealed that NF-κB activation strengthens ferroptosis via repressing the expression of solute carrier family 7 member 11(SLC7A11).^44^ Similarly, Zhang et al. found that the anti-tumor effects of thiostrepton (TST) are mediated by the inactivation of the STAT3 signaling pathway, which directly binds to the GPX4 promoter to facilitate its transcription.^45^ On the other hand, the activated STAT3 can also positively regulate SLC7A11 to attenuate the ferroptosis process in acute lung injury induced by intestinal ischemia/reperfusion.^46^ However, it has been revealed that the impairment of NF-κB/STAT3 signaling contributes to inducing ferroptosis, and is helpful to overcome drug resistance in DLBCL,^30^ which is consistent with our results. More meaningfully, our results indicated that the activation of NF-κB/STAT3 signaling was regulated by FASN. Zhuang *et al*. demonstrated that FASN inhibition promotes the radiotherapy sensitization of prostate cancer (both androgen-dependent and -independent) due to inhibition of NF-κB activity.^47^ However, the underlying mechanism remains unclear. The results presented in this study preliminarily revealed that FASN overexpression induced the phosphorylation of IKKα and IκBα, which contributes to NF-κB activation.^48^

## 5. Conclusion

The present study exhibited that FASN is deeply involved in the ADM resistance in DLBCL. Mechanistically, overexpression of FASN inhibited ferroptosis by activating the NF-κB/STAT3 signaling pathway, thereby promoting the GPX4 expression. The results in this work provide a rationale that FASN can be a promising therapeutic target for overcoming the significant issue of drug resistance in DLBCL.

## Data Availability

The data used to support the findings of this study are included within the article.
